# Implementing Enhanced Perioperative Care in Emergency General Surgery: A Prospective Multicenter Observational Study

**DOI:** 10.1007/s00268-023-06984-9

**Published:** 2023-04-06

**Authors:** Marco Ceresoli, Alan Biloslavo, Pietro Bisagni, Carlo Ciuffa, Laura Fortuna, Antonio La Greca, Dario Tartaglia, Mauro Zago, Ferdinando Ficari, Giuseppe Foti, Marco Braga, the ERAS-emergency surgery collaborative group, Francesca Teodora Armao, Andrea Bottari, Michele Ballabio, Luigi Beretta, Chiara Bondi, Serena Calcinati, Michele Carlucci, Massimo Chiarugi, Arianna Libera Ciravegna, Federico Coccolini, Valerio Cozza, Camilla Cremonini, Federica Ferraina, Valeria Fico, Michele Fogliata, Paola Germani, Luca Gianotti, Samuele Grandi, Lorenzo Guiotto, Enrico Lena, Marco Longhi, Irene Lorenzi, Alessia Malagnino, Marco Montino, Lucia Paiano, Giovanni Pesenti, Sara Riccadonna, Bruno Romanò, Andrea Russo, Riccardo Somigli, Valentina Tomajer, Vincenzo Tripodi

**Affiliations:** 1 General and Emergency Surgery Department, School of Medicine and Surgery, Milano-Bicocca University, Via Pergolesi 33, 20900 Monza, Italy; 2 General Surgery Department, Cattinara Hospital, ASUGI, Strada Di Fiume, 447, 34149 Trieste, Italy; 3 General and Emergency Surgery, ASST Lodi, Lodi, Italy; 4 General and Emergency Surgery, IRCCS Ospedale San Raffaele, Milan, Italy; 5 General Surgery, Ospedale S. Jacopo, Pistoia, Italy; 6 Emergency Surgery and Trauma, Fondazione Policlinico Universitario A. Gemelli IRCCS Roma - Universita’ Cattolica del Sacro Cuore, Rome RM, Italy; 7 General, Emergency and Trauma Surgery Unit, University of Pisa, Pisa, Italy; 8 Emergency and Robotic Surgery Department, Emergency and General Surgery Unit, A. Manzoni Hospital–ASST, Lecco, Italy; 9 General Surgery, Careggi Hospital, University of Firenze, Florence, Italy; 10 Anesthesia and Intensive Care Department, School of Medicine and Surgery, Milano-Bicocca University, Monza, Italy

## Abstract

**Introduction:**

ERAS pathway has been proposed as the standard of care in elective abdominal surgery. Guidelines on ERAS in emergency surgery have been recently published; however, few evidences are still available in the literature. The aim of this study was to evaluate the feasibility of an enhanced recovery protocol in a large cohort of patients undergoing emergency surgery and to identify possible factors impacting postoperative protocol compliance.

**Methods:**

This is a prospective multicenter observational study including patients who underwent major emergency general surgery for either intra-abdominal infection or intestinal obstruction. The primary endpoint of the study is the adherence to ERAS postoperative protocol. Secondary endpoints are 30-day mortality and morbidity rates, and length of hospital stay.

**Results:**

A total of 589 patients were enrolled in the study, 256 (43.5%) of them underwent intestinal resection with anastomosis. Major complications occurred in 92 (15.6%) patients and 30-day mortality was 6.3%. Median adherence occurred on postoperative day (POD) 1 for naso-gastric tube removal, on POD 2 for mobilization and urinary catheter removal, and on POD 3 for oral intake and i.v. fluid suspension. Laparoscopy was significantly associated with adherence to postoperative protocol, whereas operative fluid infusion > 12 mL/Kg/h, preoperative hyperglycemia, presence of a drain, duration of surgery and major complications showed a negative association.

**Conclusions:**

The present study supports that an enhanced recovery protocol in emergency surgery is feasible and safe. Laparoscopy was associated with an earlier recovery, whereas preoperative hyperglycemia, fluid overload, and abdominal drain were associated with a delayed recovery.

**Supplementary Information:**

The online version contains supplementary material available at 10.1007/s00268-023-06984-9.

## Introduction

Enhanced recovery after surgery (ERAS**®**) is an evidence-based multimodal approach to optimize perioperative pathway which allowed to reduce postoperative morbidity and shortened length of hospital stay. Therefore, enhanced recovery protocols have been proposed as the standard of care in elective colorectal surgery [1, 2].

Despite the publication of proper guidelines [3], the spread of enhanced recovery protocols in patients undergoing emergency surgery is still limited. Preliminary results have been published in patients who underwent surgery for obstructing colorectal cancer or perforated peptic ulcers [4–8]. However, systematic reviews and meta-analysis showed that enhanced recovery protocols were different across the studies, especially concerning the intraoperative items [9, 10]. Limiting factors to the widespread of enhanced recovery protocols in emergency surgery are the non-applicability of preoperative items and the presence of acute stressful diseases often requiring tailored care rather than standardized protocols [11, 12].

The aim of this study was to evaluate the feasibility of an enhanced recovery protocol in a large cohort of patients undergoing emergency general surgery and to identify possible factors impacting postoperative protocol compliance.

## Methods

This is a prospective, observational, multicenter cohort study promoted by the Italian Society of Emergency Surgery and Trauma and the Perioperative Italian Society. Eight Italian high-volume hospitals with previous experience in enhanced recovery protocols in major elective surgery were involved. The study protocol was shared during a multidisciplinary meeting involving surgeons and anesthesiologists from each center. Supplementary Table reports the study protocol which was approved by the Ethical Committee of the promoting center (n. 0,012,747 08/10/2020) and was registered on clinicaltrial.gov (identifier NCT04648644).

Patients aged   >  18 years undergoing unscheduled intestinal resections with or without anastomosis, hollow viscus injury repair, enteric bypass or adhesiolysis in presence of either peritoneal contamination or intestinal obstruction were included in the study. Exclusion criteria were refused to participate, septic shock, and emergency surgery for complications following elective surgery, operative endoscopy or diagnostic procedures. Patients presenting with multiple organ failure who required damage control surgery with open abdomen and/or immediate postoperative ICU stay longer than 72 h were dropped out from the study because they had no chance to adhere to the enhanced recovery protocol.

Demographics, body mass index, Charlson Comorbidity Index, primary diagnosis, type of surgery, adherence to each ERAS item and short-term outcome parameters were anonymously collected from all patients. Major complications have been classified according to the Clavien-Dindo scale [13]. Patients’ follow-up was carried out by office visits or telephone calls.

The primary endpoint of the study was the adherence to the enhanced recovery protocol. Secondary endpoints are 30-day mortality and morbidity rates, and length of hospital stay.

### Statistical analysis

Continuous variables were described as median and interquartile range (IQR). Categorical variables were described as percentages. The cumulative adherence was recorded daily for each postoperative item. Univariate and multiple ordinal regression models were calculated using the number of achieved postoperative items as outcome. For each postoperative item, patients’ compliance was defined using the median value as the threshold. Variables with a significant association at the univariate analysis (*p* < 0.05) were adopted in the multiple ordinal regression model. Pearson’s linear correlation was calculated between the number of achieved postoperative items and length of stay. Statistical analysis was performed using the IBM SPSS Statistics 27 software (IBM Corp. Released 2020. IBM SPSS Statistics for Windows, Version 27.0. Armonk, NY: IBM Corp).

## Results

**Fig. 1:**
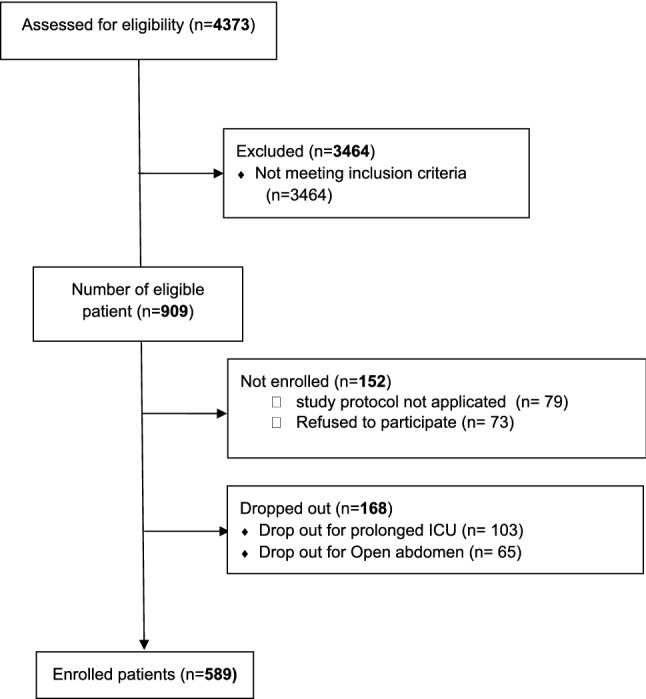
Study flow diagram

Between November 2020 and November 2021 among the eight participating centers 909 patients fulfilled the inclusion criteria. Figure 1 reports the flow diagram of the study. A total number of 589 patients were included in the analysis.

Table 1 shows that 242 (41.1%) patients were over 75 yrs., 318 (54.4%) had ASA 3–4, while body temperature was below 36.0 °C in 177 (33.5%) patients. Three hundred and twenty-seven (55.5%) patients had intestinal obstruction, while 262 (44.6%) had intra-abdominal infections. The median time from hospital admission to operation was 8 h (3–25). Two hundred and fifty-six (43.5%) patients underwent intestinal resection with anastomosis and 93 (15.8%) underwent intestinal resection without anastomosis. One hundred and forty-four (24.6%) patients were treated with minimally invasive techniques.

**Table 1:** Patients’ characteristics

		Median (IQR)	*N*	%
Age		72 (57–81)		
Age range	19–40		40	6.8%
	41–65		192	32.6%
	66–75		115	19.5%
	76–85		167	28.4%
	> 85		75	12.7%
Sex	F		311	52.8%
	M		278	47.2%
BMI		24.22 (22–26)		
BMI range	< 18		27	5.4%
	18–24		246	48.9%
	25–30		182	36.2%
	> 30		48	9.5%
ASA class	1		57	9.8%
	2		209	35.8%
	3		253	43.3%
	4		65	11.1%
Charlson comorbidity index		4 (2–7)		
Time from admission to surgery (hours)		8 (3–25)		
Time from admission to surgery (hours)	< 24 h		433	73.5%
	> 24 h		156	26.5%
pH		7.4 (7.2–7.4)		
Hb (mg/dL)		12 (11–14)		
Lac (mmol/L)		1.5 (1–2.4)		
Body temperature at admission		36.4 (36–36.9)		
Body temperature	(36.0–37.5)		318	60.2%
	(< 36.0)		177	33.5%
	(> 37.5)		33	6.3%
Preop. Glycemia (mg/dL)		120 (100–145)		
SARS-CoV2 positive nasal swab			4	0.7%
Diagnosis	Obstruction		300	51%
	Lower GI perforation		172	29%
	Ischemia		32	12%
	Incarcerated hernia		27	5%
	Upper GI perforation		48	8%
	Others		10	2%
Surgical procedure	Resection with anastomosis		256	43.5%
	Lysis of adhesion		168	28.5%
	Resection without anastomosis		93	15.8%
	Perforated peptic ulcer repair		38	6.5%
	By-pass		20	3.4%
	Hollow viscus perforation repair		14	2.4%
Surgery duration (min)		120 (80–180)		
Surgical technique	Open surgery		348	59.4%
	Laparoscopy		144	24.6%
	Laparoscopy converted to open		94	16.0%

Table 2 reports adherence to the enhanced recovery items. The highest adherence was obtained for operative warming and postoperative nausea and vomiting prophylaxis. The median operative fluid infusion was 12 (8.33–17.14) mL/Kg/h. Drains were placed in 92.7% of patients with intra-abdominal sepsis and in 55.7% of patients with intestinal obstruction.

**Table 2:** Adherence to enhanced recovery items

		Median (IQR)	*N*	%
Depth of anesthesia monitoring (entropy)			302	51.3%
Neuromuscular blockade monitoring			230	39.0%
PONV prevention			516	87.6%
General + locoregional anesthesia			142	24.1%
Active warming			551	95.8%
Invasive arterial pressure monitoring			102	17.3%
Opioid used	Fentanyl		269	47.7%
	Morphine		140	24.8%
	Remifentanil		129	22.9%
	Disufen		8	1.4%
	Ketamine		8	1.4%
	Other		10	1.8%
Intraoperative transfusion			71	12.2%
Inotropes/Vasopressors			70	12.0%
Intravenous fluids ml/kg/h		12 (8.33–17.14)		
Intravenous fluids	3–6 ml/Kg/h		125	21.2%
	7–12 ml/Kg/h		183	31.1%
	> 20 ml/Kg/h		281	47.7%
Minimally invasive surgery			144	24.6%
Drain	All patients		422	72.1%
	obstruction		181	55.7%
	Intra-abdominal sepsis		241	92.7%

*PONV*: Postoperative nausea and vomiting

Table 3 summarizes the postoperative short-term outcome. Overall morbidity was 60.4%, major complications occurred in 92 (15.6%) patients, and 30-day mortality was 6.3%. Median length of hospital stay was 8 (6–12) days.

**Table 3:** Short-term postoperative outcome

		Median (IQR)	*N*	%
Overall morbidity			356	60.44%
30-day mortality			37	6.28%
Complication grade	0		233	39.56%
	I		135	22.92%
	II		129	21.90%
	IIIa		13	2.21%
	IIIb		27	4.58%
	IVa		10	1.70%
	IVb		5	0.85%
	V		37	6.28%
Surgical site infection			82	14.16%
Anastomotic leak			23	6.97%
Respiratory infection			46	7.94%
Urinary tract infection			20	3.45%
Cardiovascular complications			63	10.90%
Readmission within 30 days			32	5.48%
Length of stay ( days)		8 (6–12)		

Table 4 shows the cumulative compliance to postoperative items. The median adherence was reached on postoperative day (POD) 1 for naso-gastric tube removal (55.3%), on POD 2 for mobilization (68.8%) and urinary catheter removal (57%), and on POD 3 for oral intake (56.4%) i.v. fluid stop (52.3%).

**Table 4:** Cumulative compliance to postoperative items

POD	0	1	2	3	4	5	6	7
Naso-gastric tube removal	31.1	55.3	77.9	88.3	92.9	95.3	97.4	98.6
Oral fluid intake	0	29.0	57.2	72.7	81.6	86.5	89.2	90.6
Mobilization > 4 h	0	36.1	68.8	85.1	92.5	96.0	97.2	97.9
Urinary catheter removal	0	29.2	57.0	71.1	78.3	83.6	86.4	88.2
Solid food intake	0	6.1	28.5	56.4	78.1	86.5	91.8	94.4
i.v. fluids stop	0	9.6	31.6	52.3	65.6	75.2	80.8	84.3

*POD*: postoperative day. i.v.: intravenous

**Fig. 2:**
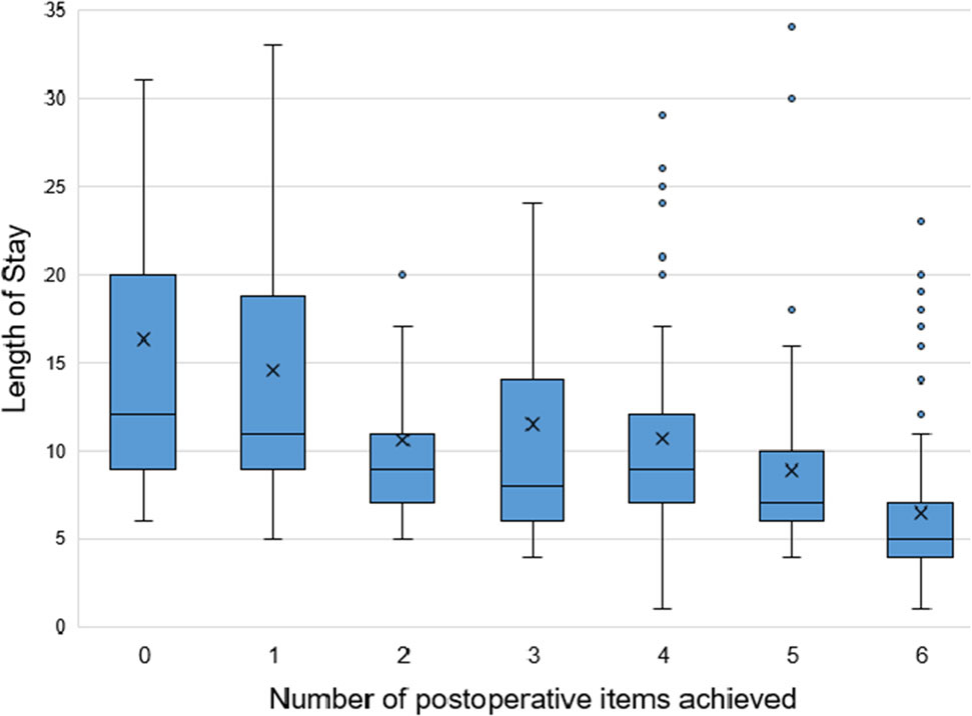
Linear correlation between number of postoperative items achieved and length of hospital stay

Table 5 shows that laparoscopy was positively associated with an increasing postoperative compliance at the ordinal logistic regression analysis. A negative association with postoperative compliance was found for preoperative hyperglycemia, operative fluid infusion > 12 mL/Kg/h, presence of abdominal drain, perforated peptic ulcer repair, duration of surgery and major complications. There was a linear correlation between the increasing postoperative items compliance and the length of hospital stay (*r* = −0.552, *p* < 0.001) as shown in Fig. 2.

**Table 5:** Univariate and multiple ordinal regression analyses for postoperative recovery items

		Univariate ordinal regression				Multiple ordinal regression			
		OR	CI 95%		.*p*-value	OR	CI 95%25		*p*-value
			Lower	Higher			Lower	Higher	
*Patients characteristics*									
Sex (men)		0.957	0.714	1.283	0.768				
Age > 70		0.445	0.330	0.599	< 0.001	0.655	0.424	1.011	0.056
BMI		1.005	0.968	1.043	0.795				
ASA III–IV		0.393	0.290	0.531	< 0.001	0.675	0.421	1.079	0.101
Charlson		0.839	0.796	0.885	< 0.001	0.922	0.844	1.008	0.076
Intra-abdominal sepsis		0.535	0.397	0.721	< 0.001	0.899	0.544	1.485	0.678
time from admission to surgery	< 24 h	1							
	> 24 h	0.731	0.524	1.021	0.066				
Preop. Glycemia		0.992	0.989	0.996	< 0.001	0.992	0.988	0.996	< 0.001
Postop. Glycemia		0.997	0.992	1.001	0.149				
pH		3.895	0.406	37.401	0.239				
Hb		1.102	1.038	1.170	0.001	1.019	0.933	1.113	0.675
Lactate		0.995	0.971	1.019	0.662				
Body temperature		0.886	0.753	1.042	0.143				
intraoperative blood transfusion		0.365	0.229	0.584	< 0.001	1.128	0.561	2.269	0.735
Inotropes/vasopressors		0.435	0.270	0.700	0.001	0.718	0.386	1.336	0.296
Duration of surgery (min)		0.994	0.992	0.996	< 0.001	0.991	0.988	0.995	< 0.001
Procedure	Lysys of adhesions	1				1			
	Bypass	0.218	0.088	0.540	0.001	0.260	0.091	1.094	0.057
	Hollow viscus perforation repair	0.479	0.191	1.202	0.117	0.507	0.159	1.619	0.252
	Resection with anastomosis	0.376	0.261	0.541	< 0.001	0.915	0.505	1.658	0.770
	Resection without anastomosis	0.308	0.191	0.495	< 0.001	1.004	0.488	2.068	0.990
	Perforated peptic ulcer repair	0.173	0.094	0.321	< 0.001	0.072	0.026	0.195	< 0.001
Major morbidity		0.292	0.185	0.460	< 0.001	0.564	0.319	0.997	0.049
*Enhanced recovery interventions*									
Depth of anesthesia monitoring (entropy)		0.956	0.714	1.282	0.765				
Neuromuscular blockade monitoring		1.649	1.217	2.236	0.001	1.240	0.830	1.852	0.293
PONV prevention		1.328	0.857	2.058	0.204				
Multimodal anesthesia		0.898	0.639	1.262	0.534				
Active warming		0.569	0.274	1.183	0.131				
Infusions ml/kg/h (continuous variable)		0.968	0.950	0.985	< 0.001				
Infusions (categorical)	[infusions 3–6 ml/Kg/h]	1				1			
	[infusions 7–12 ml/Kg/h]	0.817	0.511	1.306	0.398	0.634	0.367	1.095	0.102
	[infusions > 12 ml/Kg/h]	0.558	0.363	0.857	0.008	0.288	0.164	0.508	< 0.001
Minimally invasive surgery		3.132	2.198	4.463	< 0.001	2.222	1.395	3.538	0.001
Drain		0.277	0.195	0.393	< 0.001	0.561	0.334	0.942	0.029
Use of opioid after surgery		0.681	0.499	0.930	0.016	0.906	0.605	1.357	0.632

## Discussion

The present study supports the implementation of enhanced recovery protocols in patients undergoing emergency gastrointestinal surgery. The median adherence to postoperative recovery outcomes was reached on postoperative day 1 for naso-gastric tube removal, on day 2 for mobilization and urinary catheter removal and on day 3 for oral feeding and i.v. fluid suspension. Laparoscopy was associated with an earlier recovery, whereas preoperative hyperglycemia, fluid overload, abdominal drain, duration of surgery, and major morbidity were associated to a delayed recovery.

Few studies have been published on patients who underwent emergency surgery, the majority focused on patients with obstructing colorectal cancer or perforated peptic ulcers [4–8]. Enhanced recovery protocols were associated with shorter length of stay and lower postoperative complications when compared to traditional care. Roulin and coll. reported a lower compliance to postoperative protocol in patients who underwent urgent colectomy when compared to patients who underwent elective colectomy [6].

The present study enrolled consecutive patients without any selection bias. In fact, the majority of patients were elderly with ASA score >  2 and the median time from admission to surgery was short, thus reflecting the daily practice of emergency surgery. Patients’ compliance to postoperative items was satisfactory with all targets reached one-day later in comparison with what has been reported following elective colorectal surgery [14].

The multiple regression analysis showed that minimally invasive surgery positively impacted on postoperative recovery (*p* = 0.004). Despite a positive impact of minimally invasive technique on postoperative outcomes has been widely demonstrated in elective colorectal surgery [15, 16], the implementation of laparoscopy in emergency surgery is still a matter of debate [17, 18]. In our cohort about 40% of patients had a minimally invasive approach and laparoscopic surgery was successfully completed in two-thirds of them. A national UK study showed that laparoscopy was adopted in less than 20% of patients who underwent emergency surgery [17]. The present data should contribute to a wider use of minimally invasive approach in emergency surgery.

Intraoperative fluid management is a cornerstone of enhanced recovery protocols. In elective major noncardiac surgery a goal-directed fluid management strategy reduced postoperative complications [19, 20]. In the present study, a fluid overload negatively impacted on postoperative recovery delaying mobilization and oral feeding (*p* < 0.001). To prevent the risk of fluid overload, operative hemodynamic monitoring should be implemented to yield a proper goal-directed fluid therapy [21]. An abdominal drain was placed in 55.7% of patients operated for an intestinal obstruction, suggesting an over-indication in absence of a macroscopic peritoneal contamination. Similarly to the elective setting [22], the abdominal drain was negatively associated with postoperative recovery (*p* = 0.007), therefore, it should be placed in selected cases and removed as early as possible.

Preoperative hyperglycemia has been strongly correlated with morbidity and mortality in both elective [23–27] and critically ill surgical patients [28–30]. Hyperglycemia could also be considered a marker of severity of disease and organ dysfunction. Our data confirm the importance of a tight glycemic control in patients undergoing emergency surgery (*p* = 0.002). The repair of perforated peptic ulcer was associated with delayed postoperative recovery, too (*p* < 0.001). Despite randomized trials reported both feasibility and safety of an enhanced recovery protocol after peptic ulcer repair [4, 5], our results probably reflect some reluctance of surgeons to early feed these patients.

The present study has some limitations. Patients were recruited during the second pandemic wave with all the well-known restrictions and changes in hospital admissions and clinical practice. Pandemic affected hospitals’ organizational models making more difficult to have a dedicated equipe for emergency patients management. Moreover, this study is burdened by the intrinsic limits of the emergency setting not allowing a fixed and dedicated team with possible incomplete protocol application. On the other hand, strengths of the study are the large number of patients recruited in one-year period and the wide experience of the participating centers in enhanced recovery practice. There are several areas of possible improvement, such as preoperative hyperglycemia correction, operative fluids optimization, and implementation of minimally-invasive approach.

In conclusion, the present study supports the implementation of enhanced recovery protocols in patients undergoing emergency gastro-intestinal surgery. Multiple regression analysis showed that laparoscopy was associated with an earlier recovery, whereas preoperative hyperglycemia, fluid overload, and abdominal drain were associated with a delayed recovery.

## Supplementary Information

Below is the link to the electronic supplementary material.

Supplementary file1 (DOCX 13 KB)
