# Supplementary material for: Implementing Enhanced Perioperative Care in Emergency General Surgery: A Prospective Multicenter Observational Study
Source: World J Surg. 2023 Apr 6;47(6):1339–47. doi: 10.1007/s00268-023-06984-9 (PMC10079158; doi:10.1007/s00268-023-06984-9)
Supplement: Supplementary file 1 — Supplementary file1 (DOCX 13 KB) [file 268_2023_6984_MOESM1_ESM.docx]

Supplementary Table 1. Study protocol

| Preoperative Items: | | |
| --- | --- | --- |
| Risk assessment on admission through the Charlson Comorbidity Index | | |
| Assessment of the patient's volemic status: performing a blood gas analysis | | |
| Measurement of body temperature. | | |
| administer fluids and correct electrolytes unbalances | | |
| Early parenteral broad spectrum antibiotic therapy when appropriate | | |
| Intraoperative Items: | | |
| Anesthesia | | Prevention of PONV through dexamethasone 4 mg and/or ondasentron 8 mg |
|  |  | Multimodal anesthesia with locoregional blockage |
|  |  | Use of short acting-life opioids and hypnotics; |
|  |  | Opioid-sparing analgesia; avoid morphine |
|  |  | Active warming and body temperature monitoring |
|  |  | Monitoring depth of sedation (entropy) |
|  |  | Monitoring of neuromuscular blockade |
|  |  | Goal-directed fluid therapy and advanced hemodynamic monitoring |
|  |  | Use of crystalloid solutions: avoid 0.9% saline and synthetic colloids |
| surgery | Minimally invasive surgery whenever possible | |
|  | No drain placement unless serious peritoneal contamination | |
|  | Removal of nasogastric tube at the end of the procedure if preoperative/intraoperative stagnation <300mL and if there is no evident intestinal distension | |
| Postoperative Items: | | |
| Removal of nasogastric tube on day 0  if output <300mL or on POD 1 | | |
| Urinary catheter removal when diuresis >0.5mL/Kg/h (no oliguria) | | |
| Oral fluid intake as soon as tolerate | | |
| Solid diet resumption when fluid oral intake tolerated | | |
| Interruption of IV fluid infusion once oral fluid intake adequate | | |
| Early mobilization (at least 4h) starting from POD 1 | | |
